# Canonical, Non-Canonical and Atypical Pathways of Nuclear Factor кb Activation in Preeclampsia

**DOI:** 10.3390/ijms21155574

**Published:** 2020-08-04

**Authors:** Agata Sakowicz, Michalina Bralewska, Tadeusz Pietrucha, Dominika E Habrowska-Górczyńska, Agnieszka W Piastowska-Ciesielska, Agnieszka Gach, Magda Rybak-Krzyszkowska, Piotr J Witas, Hubert Huras, Mariusz Grzesiak, Lidia Biesiada

**Affiliations:** 1 Medical University of Lodz, Department of Medical Biotechnology, 90-752 Lodz, Poland; michalina.bralewska@gmail.com (M.B.); t.pietrucha@gmail.com (T.P.); 2 Medical University of Lodz, Department of Cell Cultures and Genomic Analysis, 90-752 Lodz, Poland; dominika.habrowska@umed.lodz.pl (D.E.H.-G.); agnieszka.piastowska@umed.lodz.pl (A.W.P.-C.); 3 Department of Genetics, Polish Mother’s Memorial Hospital-Research Institute in Lodz, 93-338 Lodz, Poland; agagach@o2.pl; 4 Department of Obstetrics and Perinatology, University Hospital in Krakow, 31-501 Krakow, Poland; rybaczka@interia.pl (M.R.-K.); huberthuras@wp.pl (H.H.); 5 Medical University of Lodz, Department of Haemostatic Disorders, 92-215 Lodz, Poland; pjwitas@gmail.com; 6 Department of Perinatology, Obstetrics and Gynecology, Polish Mother’s Memorial Hospital-Research Institute in Lodz, 93-338 Lodz, Poland; mariusz.grzesiak@gmail.com; 7 Medical University of Lodz, Department of Obstetrics and Gynecology, 93-338 Lodz, Poland; 8 Department of Obstetrics and Gynecology, Polish Mother’s Memorial Hospital-Research Institute, 93-338 Lodz, Poland; bieslidia@o2.pl

**Keywords:** canonical activation pathway, gene expression, non-canonical activation pathway, Nuclear Factor kB, placenta, preeclampsia

## Abstract

Although higher nuclear factor κB (NFκB) expression and activity is observed in preeclamptic placentas, its mechanism of activation is unknown. This is the first study to investigate whether the canonical, non-canonical, or atypical NFκB activation pathways may be responsible for the higher activation of NFκB observed in preeclamptic placentas. The study included 268 cases (130 preeclamptic women and 138 controls). We studied the expression of the genes coding for NFκB activators (NIK, IKKα, IKKβ, and CK2α) and inhibitors (IκBα and IκBβ) using RT-PCR in real time. The RT-PCR results were verified on the protein level using ELISA and Western blot. To determine the efficiency of the pathways, the ratios of activator(s) to one of the inhibitors (IκBα or IκBβ) were calculated for each studied pathway. The preeclamptic placentas demonstrated significantly lower IKKα and CK2α but higher IκBα and IκBβ protein levels. In addition, the calculated activator(s) to inhibitor (IκBα or IκBβ) ratios suggested that all studied pathways might be downregulated in preeclamptic placentas. Our results indicate that preeclamptic placentas may demonstrate mechanisms of NFκB activation other than the canonical, non-canonical, and atypical forms. In these mechanisms, inhibitors of NFκB may play a key role. These observations broaden the existing knowledge regarding the molecular background of preeclampsia development.

## 1. Introduction

Preeclampsia (PE) is a multisystem disorder that affects 5–8% of all pregnancies worldwide [1]. The clinical symptoms of PE usually appear after week 20 of gestation and are always related to the development of a new onset of hypertension in the mother. Elevated blood pressure (over 140/90 mmHg) is observed, typically accompanied by proteinuria (>300 mg for 24 h or at least 2+ on a dipstick). However, proteinuria is not required to recognize preeclampsia. According to the American College of Obstetricians and Gynecologists and the International Society for the Study of Hypertension in Pregnancy Societies, the occurrence of a new onset of hypertension combined with a new onset of thrombocytopenia, renal insufficiency, the impairment of liver function, or neurological complications is sufficient to recognize preeclampsia. Both societies divided the preeclamptic cases into early- and late-onset preeclampsia based on the time of occurrence of the disorder (i.e., before and after the 34th week of gestation) [2,3].

The nuclear factor κB (NFκB) family comprises a number of transcription factors controlling over 400 genes that play critical roles in cell activity, apoptosis, and immunity, as well as inflammation [4]. In resting cells, NFκB is found in the cytoplasm where it is linked with its inhibitor (IκB), generally IκBα or IκBβ, which are encoded by the *IKBA* or *IKBB* genes, respectively. Upon activation, a complex of NFκB subunits (e.g., p65:p50 or p65:p52) are translocated from the cytoplasm into the nucleus. There, NFκB recognises and binds to a specific DNA sequence and acts as a transcription factor [5,6].

A number of factors, including oxidants and cytokines, induce NFκB activation. This process is generally associated with IκB degradation and depends on the activators creating a multicomponent IκB kinase complex (IKK). The most common is a high molecular weight complex containing two catalytic subunits (IKKα and IKKβ encoded by the *CHUK* and *IKBKB* genes, respectively) and a scaffolding subunit of the NEMO protein, known as IKKγ, encoded by the *IKBKG* gene. This complex allows for activation of the NFκB factor by the canonical pathway, following stimulation by TNF-α, IL-1, or by angiotensin II (Ang II) [7].

Alternatively, the non-canonical pathway can be activated by B cell-activating factor (BAFF), CD40, or RNA viruses [8]. This process is IKKβ and IKKγ-independent; however, the IKKα protein, activated by the NIK protein, is required for its operation [7]. Another widely-studied NFκB pathway is the atypical pathway; this was suggested to be activated by casein kinase 2 (CK2). CK2 consists of two subunits: CK2α and CK2β, which are encoded by the *CSNK2A1* and *CSNK2B* genes, respectively. Casein kinase 2 is known to mediate IκB degradation in response to DNA damage, high levels of reactive oxygen species (ROS), or hypoxia [9,10].

The canonical, non-canonical, and atypical pathways are not only ones responsible for the activation and nuclear translocation of NFκB. Some studies suggest that the inhibitor (IκB) shuttles between the cytoplasm and nucleus, either alone or as a complex with NFκB [11,12]. In the nucleus, inhibitors may displace NFκB from the target DNA sequence and transport it back to the cytoplasm; they may also act as chaperone proteins of NFκB, allowing its persistent activation [12,13,14]. Additionally, under certain stimuli, the NFκB-IκB complex may also undergo activation in the nucleus [15,16].

The precise etiology of preeclampsia remains a mystery. Researchers proposed that insufficient trophoblast invasion of the uterine spiral arteries disturbs the process of placentation and decreases the placental perfusion resulting in its hypoxia [17,18,19]. Under unfavourable conditions, placental cells begin to produce elevated levels of inflammatory markers (e.g., IL-6 or TNF-α) and free radicals ROS, thus affecting placental function and stimulating the apoptosis of its cells [20,21,22,23]. The maternal circulation demonstrates increased levels of inflammatory markers, placental debris, and free fetal DNA released from apoptotic placental cells. These particles influence the maternal endothelial activation and dysfunction and contribute to the occurrence of the clinical symptoms of preeclampsia [20,24].

Numerous studies have linked Nuclear factor-κB with elevated levels of ROS and intensified apoptosis of placental cells [23,25,26]. A number of inflammatory markers (e.g., IL-1, IL-6, or TNF-α) observed in preeclampsia undergo upregulation by NFκB. Therefore, it is not surprising that preeclampsia cases are characterised by hyperactivation of NFκB, as well as its elevated levels in the placenta and in maternal circulation [27,28,29,30].

However, little is known regarding the mechanisms, or pathways, responsible for the presence of the active form of NFκB in the nucleus. Such an understanding may hasten the discovery of new therapeutic targets and the development of new drugs.

In the present study, we examine whether preeclamptic and non-complicated pregnancies differ with regard to the gene expression and protein levels of activators and inhibitors known to play a role in NFκB activation. The calculated ratios of activator(s) to one of the inhibitors (IκBα or IκBβ) implicated in the canonical, non-canonical, or atypical NFκB regulation pathways might indicate which of the analysed pathways should be considered the most important in the pathogenesis of preeclampsia.

## 2. Results

### 2.1. Characterization of Study Population

The clinical and laboratory characteristics of all patients qualified for the study and control groups, as well as the perinatal outcomes, are presented in Table 1.

Significant differences were found between the groups for hematocrit, red blood cell (RBC) volume, mean corpuscular hemoglobin concentration, and platelet number. The preeclamptic group demonstrated a slightly higher BMI; however, their newborns were shorter and lighter than those of the controls.

### 2.2. Preeclamptic Placentas Differ in the Expression Level of Activators and One of the Inhibitors of NFκB from Non-Complicated Cases

All NFκB activators implicated in the canonical, non-canonical, and atypical pathways, apart from the *MAP3K14* gene coding for the NIK protein, demonstrated lower expression in the population of preeclamptic women (*p* < 0.001). Additionally, preeclamptic placentas also demonstrated an elevated *IKBA* inhibitor profile (*p* = 0.004). However, fold changes (FC) for the *CSNK2A1* and *IKBA* genes failed the set criteria outlined in the Statistics section; thus, the results should not be considered significant. The results of the gene expression analyses are given in Table 2, together with the NFκB activator(s) to inhibitor ratios for the three studied pathways.

The results demonstrated that placental tissue differed in the expression of genes coding for activators (*IKKα*, *IKKβ*, and *CK2α*) and the *IκBα* inhibitor between preeclamptic and non-complicated pregnancies. The profiles of NFκB activation calculated by the ratio of activator(s) to the inhibitor typical for the studied pathways were significantly depleted for preeclamptic placentas.

### 2.3. The Protein Levels of IκBα and IκBβ Inhibitors Were Higher, Whereas IKKα and CK2α Activators Were Lower in Preeclamptic Placentas

The concentrations of each studied activator and inhibitor are presented in Table 3.

**Figure 1:**
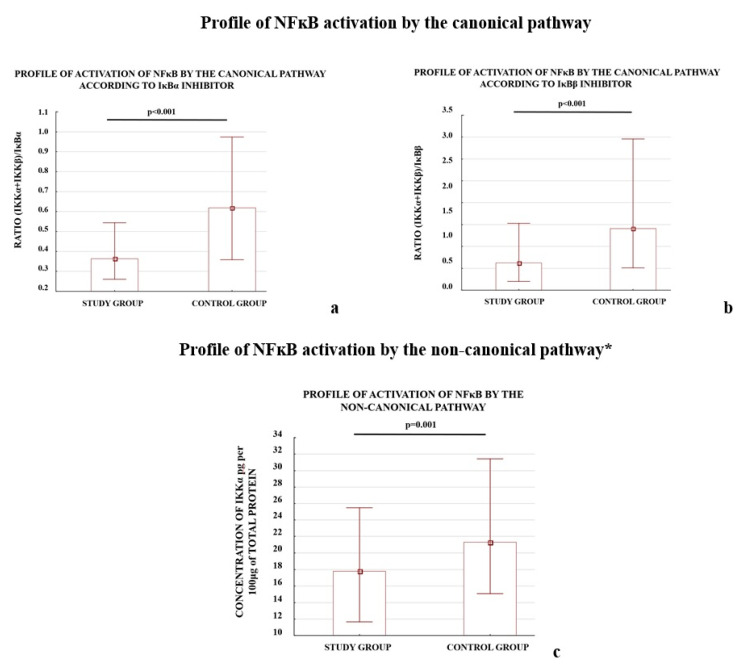

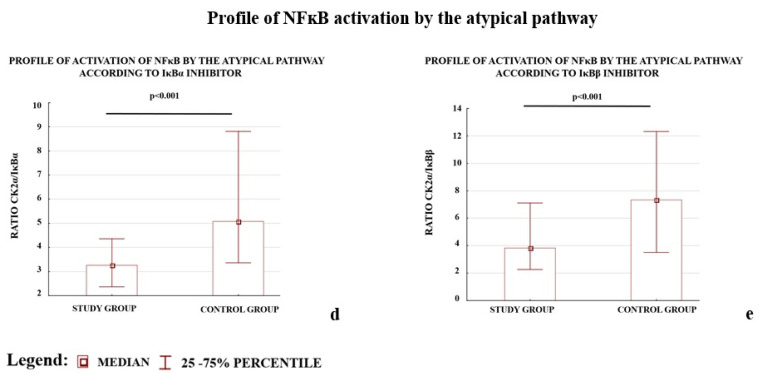
Comparison of profile of NFκB activation by the canonical (**a**,**b**), non-canonical (**c**), and atypical (**d**,**e**) pathways. * The profile of the non-canonical pathway is calculated only according to the results for the IKKα protein; the NIK protein was not tested because it does not directly activate NFκB, instead it works through IKKα. Comparison between the study (*N* = 114) and control (*N* = 133) groups; Mann–Whitney *U*-test; a *p*-value of <0.05 indicates significant results.

We also observed that the profiles of NFκB activation calculated as the ratio of activator(s) to the one of the inhibitors (IκBα or IκBβ) for the three studied pathways differed between the study and control groups (Figure 1).

The early (<34 week of pregnancy) and late (≥34 week of gestation) preeclamptic subgroups demonstrated significantly lower ratios for the canonical and atypical pathways of NFκB activation according to the controls. The *p*-value for each comparison was below 0.001; except one: the (IKKα + IKKβ)/IκBβ ratio calculated for early preeclampsia and controls, differed significantly (*p*-value 0.002). The IKKα protein level, which represents the non-canonical pathway, independent of other studied activators, differed significantly between early and late preeclampsia as well as between late preeclampsia and the controls.

The results demonstrated that both the depletion of the NFκB activators, IKKα and CK2α, and elevation of the NFκB inhibitors, IκBα and IκBβ, were characteristic of preeclamptic placentas. A reduction in the levels of the calculated ratios for all analyzed NFκB activation pathways between the study and control groups was observed.

### 2.4. Western Blot Results Confirmed the ELISA Observations; the Levels of CK2α and IKKα Were Depleted, whereas IκBα and IκBβ Were Elevated in Preeclamptic Placentas

**Figure 2:**
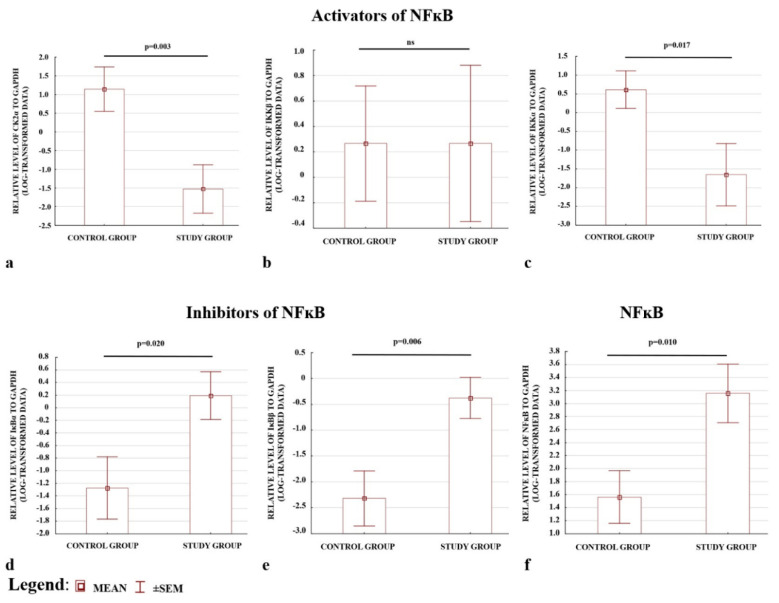
Comparison of the relative levels of activators (**a**–**c**) and inhibitors (**d**,**e**) of NFκB analysed by Western blot. (**f**) Western blotting; NFκB protein expression normalized to GAPDH. All data are presented as means ± SEM for log-transformed data. Comparison between the study (*N* = 29) and control (*N* = 29) groups; Mann–Whitney *U*-test; a *p*-value of <0.05 indicates significant results; ns—not significant.

The preeclamptic placentas were characterised by lower levels of activators, apart from IKKβ, (Figure 2a–c), and higher levels of inhibitors (Figure 2d,e); (see also Figure S1). The placentas also demonstrated higher p65 protein levels in the total fraction compared with the controls. Hence, a higher level of NFκB protein was present in preeclamptic pregnancies (Figure 2f). The results confirmed the ELISA observations that the levels of the CK2α and IKKα activators were depleted, whereas the IκBα and IκBβ inhibitors of NFκB were elevated in preeclamptic placentas.

## 3. Discussion

Researchers have postulated that preeclampsia may develop in response to the improper implantation of the trophoblast cells into the maternal decidua. Shallow trophoblast invasion leads to hypoxia, the generation of reactive oxygen species (ROS) and inflammatory markers, as well as apoptosis [22,31]. NFκB is engaged in all of these processes [32,33,34]. 

A considerable body of evidence suggests that NFκB demonstrates elevated expression and activation both in maternal cells and in preeclamptic placentas [25,29,35]. However, no information is currently available regarding the molecular mechanisms of NFκB activation. We postulate that the knowledge regarding NFκB in the placenta during normal and pathological pregnancies allows for a better understanding of human placentation as well as the molecular background of preeclampsia. This knowledge would also aid in the process of identification of new therapeutic targets for preeclampsia [27].

**Figure 3:**
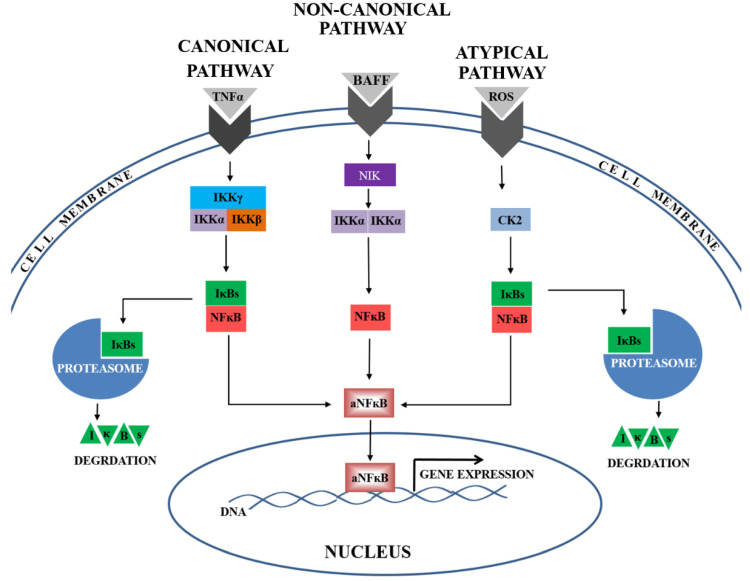
The canonical, non-canonical, and atypical NFκB signaling pathways are activated by multiple stimulants, e.g., TNF-α, BAFF or ROS. In the canonical pathway, the complex of activators, including: IKKγ, IKKβ, and IKKα, is responsible for NFκB activation by proteolysis one of the following inhibitors (IκBs): IκBα or IκBβ. In the non-canonical pathway, IKKα, supported by NIK, is responsible for NFκB activation. In the atypical pathway, the casein kinase 2 (CK2) mediates IκB (IκBα or IκBβ) degradation in the proteasome. Following the activation, NFκB is rapidly translocated into the nucleus. There, the active form of NFκB (aNFκB) recognizes the consensus sequence in the DNA and regulates the expression of numerous genes.

At present, the most widely studied pathways of NFκB activation are the canonical, non-canonical, and atypical ones (Figure 3). Our present findings indicate the presence of lower activator levels (IKKα and CK2α) and higher inhibitor levels (IκBβ and IκBα) during the canonical, non-canonical, and atypical NFκB activation pathways. In addition, the calculated ratios of activator(s) to inhibitor level were depleted in preeclamptic placentas, suggesting that all three analysed NFκB activation pathways might be down-regulated. Taken together, these findings may imply that some unknown mechanism of NFκB activation is responsible for preeclampsia development.

Only one study has examined the process of NFκB activation in both the decidua and the villous trophoblasts from the first trimester of pregnancy. Researchers found that NFκB is generally activated at the beginning of the pregnancy by the action of IKKα and IKKβ proteins, and the process is gradually down-regulated over the course of gestation to allow for the development of an immunosuppressive mechanism necessary for the maintenance of pregnancy [36].

NFκB inhibitors are degraded by the combined action of IKKα and IKKβ, together with IKKγ, thus, allowing the transcription factor to be translocated into the nucleus. The NEMO protein is known to be depleted in preeclamptic placentas [37,38], suggesting that the canonical pathway is disturbed. Our present findings further support this hypothesis. All proteins engaged in canonical NFκB pathway activation, except for IKKβ, were found to be dysregulated in preeclamptic placentas, with the activator (IKKα) being diminished and the inhibitors elevated. In addition, preeclamptic pregnancies demonstrated significantly lower activators to inhibitor ratios, (IKKα + IKKβ/IκBα) or (IKKα + IKKβ/IκBβ), compared to the controls.

As IKKα also acts as a key activator of NFκB in the non-canonical pathway, these findings also suggest that this pathway might be disturbed in preeclamptic placentas. Interestingly, the role of IKKα depends on its localisation, i.e., activating NFκB in the cytoplasm and terminating NFκB-mediated gene expression in the nucleus [39]. Therefore, a depletion in the cellular IKKα level may influence the nuclear transcriptional activity of NFκB, leading to elevated concentrations of factors whose genes are regulated by NFκB. This may, in some way, account for the elevated production of certain inflammatory factors that is observed in preeclamptic placentas, in particular TNF-α, IL-6, or IL-1β [40,41,42,43]. Of these, TNF-α and IL-6 have been found to be elevated in hypertensive rats presenting low IKKα expression [44].

The nuclear localisation of IKKα is also known to influence cell cycle progression, cell proliferation, and apoptosis [39]. All of these processes also involve CK2, the key activator of the atypical NFκB activation pathway [45]. CK2 downregulation has been found to play a significant role in inducing apoptotic processes in cancer cells, especially after induction by TNF-α, and to exacerbate the oxidative stress characteristic of preeclamptic placentas [46,47,48,49]. In addition, in vitro studies on trophoblastic cell lines indicate that hypoxia, also characteristic of preeclamptic placentas, can also down-regulate CK2 expression, thus disturbing trophoblast invasion, migration, and syncytialization, all of which are dysregulated in preeclampsia [50,51,52,53].

The results of the present study indicate that both the gene expression of casein kinase 2 alpha (the catalytic subunit of CK2 protein) and its placental concentration are down-regulated in preeclampsia. However, while a previous study found CK2α to be up-regulated in preeclamptic pregnancies, the control group used in the study was not representative of non-complicated pregnancies as it consisted of women who delivered prematurely for reasons other than preeclampsia [50]. The low ratios of CK2α to IκBα or CK2α to IκBβ observed in the present study suggest that the third most common pathway of NFκB activation in preeclamptic placentas is also dysregulated.

All estimated ratios, calculated separately for the early and late subpopulations of preeclampsia, except for those of the non-canonical pathway, remained lower than the control values. This implies that both the canonical and atypical pathways might be important for the development of preeclampsia, irrespective of the time of appearance of symptoms. Additionally, only placentas from late-onset preeclampsia were characterised by a significant reduction in the IKKα level compared to both the control and early preeclamptic samples. This might suggest that the non-canonical pathway is important for late preeclampsia development and both types of preeclampsia differ slightly according to their molecular mechanism, as postulated previously [54]. However, the time of delivery might also influence the observed results. As the week of labour between late preeclampsia and the control was similar (38 vs. 39), significant differences were observed between the early PE (32nd week) and late PE (38th week) and controls (39th week). The interpretation of the results is complicated by the lack of a sufficient reference group for early preeclampsia. This issue is further discussed in the limitations of the present study.

Our findings suggest that preeclamptic placentas may be characterised by an unknown mechanism of NFκB activation, which allows it to be maintained in an active state in the nucleus. One possibility is that the NFκB inhibitors whose levels were found to be elevated in this study may favour NFκB activation by switching from an inhibitory role to a chaperone-like function, and thus supporting the transport of NFκB into the nucleus [13]. Under the influence of some stimulators, it is possible that NFκB activation may take place independent of its typical activators, i.e., IKKα, IKKβ, IKKγ, and CK2; however, this would depend on cooperation between p53 and the RSK1 proteins [15,16,55].

A number of studies suggest that the p53 protein level is typically elevated in preeclamptic placentas, and that this is responsible for the induction of placental cell apoptosis [56,57,58]. It is possible that NFκB activation may be facilitated by cooperation between p53 and RSK1. It is also possible that p53-induced NFκB activation is involved in p53-induced programmed cell death [15]. This process of NFκB activation by p53-RSK1 takes place independently from IκBα proteosomal degradation in the cytoplasmatic compartment; however, IκBα is needed to allow the inactive form of NFκB to enter the nucleus. There, under the influence of p53-RSK1, IκBα is removed from the NFκB-IκBα complex, liberating the active form of NFκB. Therefore, the presence of an elevated level of IκBα, observed in this study, may support the migration of NFκB into the nucleus and, thus, its activation, assuming that the pathway exists in preeclamptic placentas.

The binding of NFκB with DNA is also supported by the inhibitor IκBβ, which was also found to be present in significantly greater amounts. Suyang et al. [13] reported that IκBβ may form a stable complex with NFκB in the cytoplasm. This complex, similar to NFκB-IκBα, migrates to the nucleus, where it binds to DNA and creates the IκBβ-NFκB-DNA complex, which activates gene expression [13,59]. After binding to DNA, part of IκBβ is degraded; however, the NFκB-DNA complex can still regulate gene expression, even with the loss of IκBβ. One of the genes regulated by NFκB codes for the IκBα protein; the newly-synthesised IκBα is believed to bind to NFκB in cytoplasm or enter the nucleus and displace NFκB from the DNA [13,60,61]. However, in cells subjected to prolonged stimulation, the IκBβ-NFκB-DNA complex is stable and resistant to elevated levels of IκBα [13].

Further studies are needed to confirm whether the inhibitors of NFκB may switch their inhibitory nature to a chaperone-like function in preeclamptic placentas. They should also examine whether the nuclear activation of NFκB is dependent on p53 and RSK1 in placental cells.

One of the main strengths of this study is that it examined a large population of women who developed preeclampsia without the effect of diseases or risk factors, thus eliminating population heterogeneity and laboratory bias. The present study has also some limitations. The first main limitation of the study is that as it is impossible to collect control placentas delivered at the same time as these from early preeclampsia, the control group is composed of heathy patients who delivered by caesarean section at term. This group is not suitable for comparing results between early preeclampsia and non-complicated pregnancies. Forming control groups from placentas delivered prematurely due to reasons other than preeclampsia may also have a significant effect on the results. The present study evaluates both the expressions of the genes and the protein levels associated with the three most well-studied molecular pathways of NFκB activation. Although the concentrations of the activators were depleted and the inhibitors were elevated, the activity of the studied proteins was not estimated (e.g., by using the antibodies against activation loop phosphorylation). Hence, the present results suggest that all three canonical, non-canonical, and atypical pathways are down-regulated in preeclamptic placentas. Further functional studies are warranted to estimate the activity of studied pathways characteristic of preeclampsia.

## 4. Materials and Methods

### 4.1. Study and Control Groups

The study included 268 women in single pregnancy (130 preeclamptic women and 138 controls). The study protocol was approved by the Medical University of Lodz Ethical Committee (RNN/20/18/KE from 16th of January 2018), and all procedures followed the Declaration of Helsinki principles. Informed consent for the collection of biological material and its use for further analyses was obtained from all patients before delivery.

Preeclampsia was diagnosed on the basis of the following symptoms: maternal blood pressure over 140/90 mmHg (measured twice with an interval of at least six hours) accompanied by proteinuria (>300 mg/24 h or at least 2+ during a single urine test), which developed after week 20 of gestation. Patients with early (<34 week of pregnancy; *n* = 55) and late (≥34 week of gestation; *n* = 75) preeclampsia were both qualified for the study group. Women qualified for the study group delivered by caesarean section.

The control group included healthy patients who delivered by caesarian section due to transverse or breech position of the fetus, ophthalmological indications, orthopedic indications, or an increased risk of uterine rupture due to a previously performed caesarean section.

The following exclusion criteria were used for all participants: other than single pregnancy, chronic diseases, gestational diabetes mellitus, hypertension diagnosed before pregnancy, maternal BMI before pregnancy >30 kg/m^2^, fetal chromosomal abnormalities, and uterus activity, which signals delivery.

### 4.2. Collection of Placental Samples

Immediately after birth, the placenta was trimmed approximately 5 cm from the site of the umbilical cord insertion into the placenta. The decidua and amnion were removed and the placental samples were immediately washed in sterile phosphate buffered saline (PBS) to remove excess blood.

In the next step, the placental fragment used in the present study was set in RNAlater (Ambion Inc., NY, USA) to protect the RNA and protein from degradation. Following 8–12 h of incubation at 4 °C in RNAlater, the placental tissue was cut into small pieces, which were stored at −20 °C or −80 °C.

### 4.3. Gene Expression Analysis

The placental tissue samples stored at −20 °C were used for total RNA isolation using the Total RNA Mini Kit according to the manufacturer’s instructions (A&A biotechnology, Gdynia, Poland). The concentration and purity of the RNA was determined spectrophotomerically (NanoDrop, Thermo Fisher Scientific, Grand Island, NY, USA) and 2000 ng of pure RNA (OD_260/280_ between 1.8–2.0) was transcribed into cDNA using a Maxima First Strand cDNA kit (Thermo Fisher Scientific, Waltham, MA, USA). The expression of the selected genes (Table 4) was analysed using commercial RT2 Profiler PCR Assays (Qiagen GmbH, Hilden, Germany) according to the manufacturer’s instructions in a LightCycler 480 (Roche, Mannheim, Germany) device.

The reactions were normalised according to GAPDH and YWHAZ as housekeeping genes, and to a reference sample including pooled cDNA from the study and control groups. Normalized gene expression was calculated as described by Pfaffl [62]. The results for relative gene expression were confirmed based on a measurement of absolute gene expression performed using a Droplet Digital^TM^ PCR System (Bio-Rad Laboratories, Hercules, CA, USA). The same RT2 Profiler PCR Assays from Qiagen were adopted for these analysis.

### 4.4. Protein Isolation

Pieces of placental tissue, of approximately 0.1 mg, were washed in PBS and homogenized with 1000 μL of PBS pH 7.2 supplemented by 1× protease and phosphatase inhibitor cocktail. The homogenates underwent three freeze–thaw cycles comprising freezing at −80 °C for 10 min followed by thawing. The samples were then passed through a 26-gauge needle 10 times then centrifuged at 14,000 rpm for 10 min at 4 °C. Following the centrifugation, the supernatant rich total protein fraction was collected. The concentration of the placental total protein fraction was detected using the BCA method. The protein fraction was divided into small portions, and each was stored at −80 °C for future analysis.

### 4.5. ELISA Analyses

We performed the quantitative determination of the IKKα, IKKβ, CK2α, IκBα, and IκBβ proteins in the placental total protein fractions using Enzyme Linked-Immunosorbent Assay (ELISA). All procedures related to ELISA were performed according to the manufacturer’s instructions (Cloud Clone, Wuhan, China). The dilution factors for each analysed protein were experimentally estimated as follows: 50-fold for CK2α, 100-fold for IKKα, 200-fold for IKKβ, 10-fold for IκBα, and 5-fold for IκBβ. Briefly, 100 μL aliquots of the diluted samples and calibrators were placed in the wells of a ready-to-use microplate, which was commercially pre-coated with the specific antibody against the analysed protein. The microplate was incubated for one hour at 37 °C. The liquid was then removed from the plate, and 100 μL of Detection Reagent A (including the biotin-conjugated antibody against the analyzed protein) was added. Following one hour of incubation at 37 °C, the liquid was aspirated, and each well of the microplate was washed three times using the wash solution. Next, 100 μL of avidin conjugated to horseradish peroxidase (HRP) was added to each well. After a half hour of incubation, the microplate was washed five times and incubated with 90 μL of the substrate solution (TMB) for 10 min at 37 °C. The avidin exhibited a color change from transparent white to blue. The enzyme–substrate reaction was terminated by the addition of the stop solution, yielding a blue color in each well. The absorbance was measured at 450 nm.

The intra- and inter-assay precisions, given as coefficient variation (CV%), were found to be below 17% for all tests, which is in line with the FDA recommendations [64]. The ELISA results were recalculated accordingly: nanograms (ng) or picograms (pg) of studied protein per 100 μg of total protein isolated from the placental sample.

### 4.6. Western Blot Analysis

The total proteins were randomly pooled (10–12 samples together) in various combinations to prepare the following samples: control (*N* = 29), early (*N* = 13), and late preeclampsia (*N* = 16). Western blot was used to verify the ELISA results and to check the status of p65 (NFκB) in the placentas.

Briefly, 30 μg aliquots of the total protein were loaded onto 10% SDS-PAGE and separated under reducing conditions at 100 V. Following the electrophoresis, the proteins were transferred to a PVDF microporous membrane (Millipore, Billerica, MA, USA). In the next step, the membrane was cropped according to the protein ladder, and all fragments were blocked for one hour in 5% non-fat milk. The fragments were then incubated overnight with one of the following primary antibodies diluted in 1% BSA in TBST buffer: IKKα (MAF-16157), IKKβ (LF-MA0192), CK2α (PA5-28686), IκBα (PA5-22120), IκBβ (MA5-15673), and p65 (MA5-15563). The dilution factor for each antibody was as follows: 1:500 for CK2α, 1:1000 for IKKα, 1:2000 for IKBβ, 1:5000 for IκBα, 1:500 for IκBβ, and 1:2000 for p65. The monoclonal anti-GAPDH antibody (MA5-15738) was used as the internal control. Following this, the membranes were washed three times in TBST and incubated for one hour with the mouse or rabbit secondary antibodies conjugated with HRP. The proteins were visualised in a chemiluminescence device (ChemiDoc, Bio-Rad Laboratories, Hercules, CA, USA). The optical density of the bands was analyzed using ImageLab software (Bio-Rad Bio-Rad Laboratories, Hercules, CA, USA).

### 4.7. Statistical Analyses

Statistical analyses were performed using Statistica version 13.1 (Statsoft, Krakow, Poland). Normally distributed data were presented as the mean ± standard deviation (SD) and analysed with Student’s *t*-test; non-normally distributed data were given as the median and interquartile range and tested with the Mann–Whitney *U*-test. The categorical variables were analysed with the chi-square test or Yates’s correction. The ELISA outcomes were compared between the early preeclampsia, late preeclampsia, and control groups using the Kruskal–Wallis test with the *post hoc* Dunn’s test. The western blot data were log-transformed and compared statistically using Student’s *t*-test. The efficiency of the NFκB activation pathways was assessed based on the gene expression and ELISA results. The efficiency was calculated for the following ratios: IKKα+IKKβ/IκBα and IKKα+IKKβ/IκBβ for the canonical pathway; IKKα for the non-canonical pathway; and CK2α/IκBα and CK2α/IκBβ for the atypical pathway. All reported tests were two-tailed, and a *p*-value below 0.05 for the test results was considered statistically significant. The gene expression results were considered as significant when both the *p*-value was <0.05 and the fold change (FC) was >1.5 or <−1.5.

## 5. Conclusions

Our findings indicated that the calculated ratios of activators to inhibitor for each of the most common NFκB activation pathways, namely, the canonical, non-canonical, and atypical pathways, are reduced in preeclamptic placentas. In these pathways, the levels of activators, such as IKKα and CK2α, were depleted, whereas the levels of both inhibitors (IκBα and IκBβ) were elevated. Only the IKKβ activator did not differ between the preeclamptic and control groups. This suggests that all studied pathways may be down-regulated in preeclamptic placentas. As numerous studies have reported that NFκB demonstrated higher expression and activity in preeclampsia, it is possible that another mechanism of NFκB activation typical of preeclampsia may exist. In this mechanism, the inhibitors may switch to functioning as chaperone-like proteins, thus, enabling the nuclear translocation of NFκB. In the nucleus, NFκB may undergo activation by other regulators or, together with an inhibitor, may bind to DNA and commence the transcription of numerous genes implicated in preeclampsia development.

## Figures and Tables

**Table 1:** Clinical parameters of mother and their children enrolled in the study.

Parameters	Study Group *N* = 130	Control Group *N* = 138	*p*
Maternal age at the time of delivery (years) ^1^	31 (27–35)	32 (30–35)	0.056
WBC (10^3^/uL) ^1^	10.1 (8.9–12.1)	10.1 (8.9–11.7)	0.699
RBC (10^6^/uL) ^1^	4.2 (3.8–4.4)	4.2 (3.9–4.4)	0.365
HB (g/dL) ^1^	12.4 (11.3–13.1)	12.5 (11.8–13.1)	0.298
HCT (%) ^2^	35 ± 3.3	36 ± 2.7	0.011
MCV (fL) ^1^	85.5 (83.3–88.0)	87.2 (84.0–90.9)	0.003
MCHC (g/dL) ^1^	34.7 (33.8–35.3)	34.1 (33.2–34.9)	<0.001
PLT (10^3^/uL) ^2^	195 ± 63	221 ± 55	<0.001
BMI (kg/m^2^) ^1^	26 (24–29)	24 (22–28)	<0.001
Newborn weight (g) ^1^	2130 (1500–3100)	3370 (3100–3710)	<0.001
Newborn length (cm) ^1^	48 (42–52)	54 (52–56)	<0.001
Week of delivery (week) ^1^	32 (29–33) (early preeclampsia) 38 (36–39) (late preeclampsia)	39 (38–39)	<0.001 <0.001
Primiparous n (%) ^3^	83 (64%)	47 (34%)	<0.001
History of miscarriage n (%) ^3^	23 (18%)	25 (18%)	0.928
Male sex of the fetus n (%) ^3^	74 (57%)	69 (50%)	0.256

Legend: BMI, body mass index; WBC, white blood cells; RBC, red blood cells; HB, hemoglobin concentration; HCT, hematocrit; MCV, mean corpuscular volume; MCHC, mean corpuscular hemoglobin concentration; PLT, platelets; kg/m^2^, kilograms/meter square; µL, microliter; g/dL, grams/deciliter; %, percent; fL, fentoliter; g, grams; cm, centimeter; n, number of cases; ^1^ non-normal distributed data, p value calculated by the Mann–Whitney U-test; ^2^ normal distributed data, *p*-value calculated by Student’s *t*-test; ^3^ categorical data, *p* value calculated by *chi*2 or *chi*2 with Yates correction tests.

**Table 2:** Comparison of gene expression between the study and control groups.

Gene Symbol or Ratio	Study Group Median * (Interquartile Range) *N* = 130	Control Group Median * (Interquartile Range) *N* = 138	Fold Change	*p* **
**NFκB activators for the canonical, non-canonical and atypical pathways**				
*CHUK*	1.42 (0.81–2.45)	2.38 (1.22–4.12)	−1.68	<0.001
*IKBKB*	0.88 (0.45–1.66)	1.44 (0.94–2.43)	−1.64	<0.001
*CSNK2A1*	1.07 (0.71–1.54)	1.52 (0.97–2.46)	−1.42	<0.001
*MAP3K14*	1.48 (0.51–3.56)	0.92 (0.50–3.26)	1.61	0.393
**NFκB inhibitors**				
*IΚBA*	1.32 (0.83–1.78)	1.07 (0.76–1.38)	1.23	0.004
*IKBB*	1.02 (0.60–1.89)	0.84 (0.53–1.71)	1.21	0.098
**Profile of NFκB activation by the canonical pathway**				
*(CHUK+IKBKB)/IΚBA*	2.14 (1.27–4.28)	3.80 (2.60–7.10)	−1.78	<0.001
*(CHUK+IKBKB)/IKBB*	2.34 (1.05–4.19)	4.29 (2.15–8.87)	−1.83	<0.001
**Profile of NFκB activation by the non-canonical pathway**				
CHUK ***	1.42 (0.81–2.45)	2.39 (1.22–4.12)	−1.68	<0.001
CHUK+MAP3K14 ***	3.47 (1.87–5.91)	3.44 (1.96–8.15)	1.00	0.328
**Profile of NFκB activation by the atypical pathway**				
*CSNK2A1/IΚBA*	0.86 (0.53–1.47)	1.62 (0.99–2.58)	−1.88	<0.001
*CSNK2A1/IKBB*	1.11 (0.50–1.89)	1.66 (0.80–3.23)	−1.50	<0.001

* The median and interquartile range were calculated based on relative gene expression calculated by the Pfaffl method; ** *p* value calculated by the Mann–Whitney *U*-test. *** In the non-canonical pathway, only the IKKα protein (encoded by the *CHUK* gene) is directly engaged in NFκB activation; the NIK protein (encoded by the *MAP3K14* gene) is necessary for stimulation of IKKα; therefore, IKKα represents the power of activation of NFκB by the non-canonical pathway.

**Table 3:** Comparison of the concentration of activators and inhibitors of NFκB between the study and control group.

Gene Symbol	Study Group Median ^1^ (Interquartile Range) *N* = 114	Control Group Median ^1^ (Interquartile Range) *N* = 133	*p* ^2^
**NFκB activators for the canonical, non-canonical and atypical ^3^ pathway**			
IKKα **	17.8 (11.6–25.5)	21.3 (15.1–31.4)	0.001
IKKβ *	11.6 (9.1–15.9)	13.1 (8.7–22.0)	0.147
CK2α **	255.6 (191.3–403.5)	343.1 (238.6–456.0)	<0.001
**NFκB inhibitors**			
IκBα **	84.2 (59.3–109.7)	64.0 (39.9–91.2)	<0.001
IκBβ **	64.9 (39.1–99.5)	51.0 (30.6–78.3)	0.004

^1^ The median and interquartile range were calculated based on the results of concentration [ng] * or [pg] ** of the studied protein after correction according to 100 μg of total protein isolated from placenta ^2^ *p* value calculated by the Mann–Whitney U-test; ^3^ The concentration of the NIK protein, one of the activators of NFκB, was not studied because this protein does not activated directly NFκB but works by IKKα.

**Table 4:** Selected gene implicated in NFκB regulation pathways and candidate housekeeping genes for placental samples.

Unigene	GeneBank	Gene Symbol/ (Protein Symbol)	The Full Name of the Gene/Protein
**NFκB activators for the canonical, non-canonical and atypical pathways**			
Hs.198998	NM_001278	*CHUK/* (IKKα) ^1,2^	Conserved Helix-loop-helix Ubiquitous Kinase or Inhibitor of Nuclear Factor Kappa-B Kinase Subunit Alpha/I-Kappa-B-Kinase Alpha
Hs.597664	NM_001190720	*IKBKB/* (IKKβ) ^1^	Inhibitor of Nuclear Factor Kappa B Kinase Subunit Beta I-Kappa-B-Kinase Beta
Hs.644056	NM_001895	*CSNK2A1/* (CK2α) ^3^	Casein Kinase 2 Alpha 1
Hs.404183	NM_003954	*MAP3K14/* (NIK) ^2^	Mitogen-Activated Protein Kinase Kinase Kinase 14/NF-Kappa-Beta-Inducing Kinase
**NFκB inhibitors**			
Hs.81328	NM_020529	*IKBA*(IκBα) ^1,3^	Nuclear Factor of Kappa Light Polypeptide Gene Enhancer In B-Cells Inhibitor, Alpha/NF-Kappa-B Inhibitor Alpha
Hs.9731	NM_001001716	*IKBB*(IκBβ) ^1,3^	Nuclear Factor of Kappa Light Polypeptide Gene Enhancer In B-Cells Inhibitor, Beta/NF-Kappa-B Inhibitor Beta
**Housekeeping gene candidate for placental samples**			
Hs.520640	NM_001101	*ACTB*	Actin Beta
Hs.544577	NM_001256799	GAPDH *	Glyceraldehyde-3-Phosphate Dehydrogenase
Hs.546285	NM_001002	*RPLP0*	Ribosomal Protein Lateral Stalk Subunit P0
Hs.520348	NM_021009	*UBC*	Ubiquitin C
Hs.492407	NM_001135699	YWHAZ *	Tyrosine 3-Monooxygenase/Tryptophan 5-Monooxygenase Activation Protein Zeta

* The best layout of housekeeping genes according to NormFinder [63]; ^1,2,3^ The product of genes engaged in canonical ^1^, non-canonical ^2^, and atypical ^3^ pathways.

## Supplementary Materials

The following are available online at https://www.mdpi.com/1422-0067/21/15/5574/s1. Figure S1: Activators and inhibitors of NFĸB in Western blot visualization.
