# Supplementary material for: Canonical, Non-Canonical and Atypical Pathways of Nuclear Factor кb Activation in Preeclampsia
Source: Int J Mol Sci. 2020 Aug 4;21(15):5574. doi: 10.3390/ijms21155574 (PMC7432517; doi:10.3390/ijms21155574)
Supplement: Supplementary file 1 [file ijms-21-05574-s001.pdf]

Supplementary Figures – Sakowicz et al.

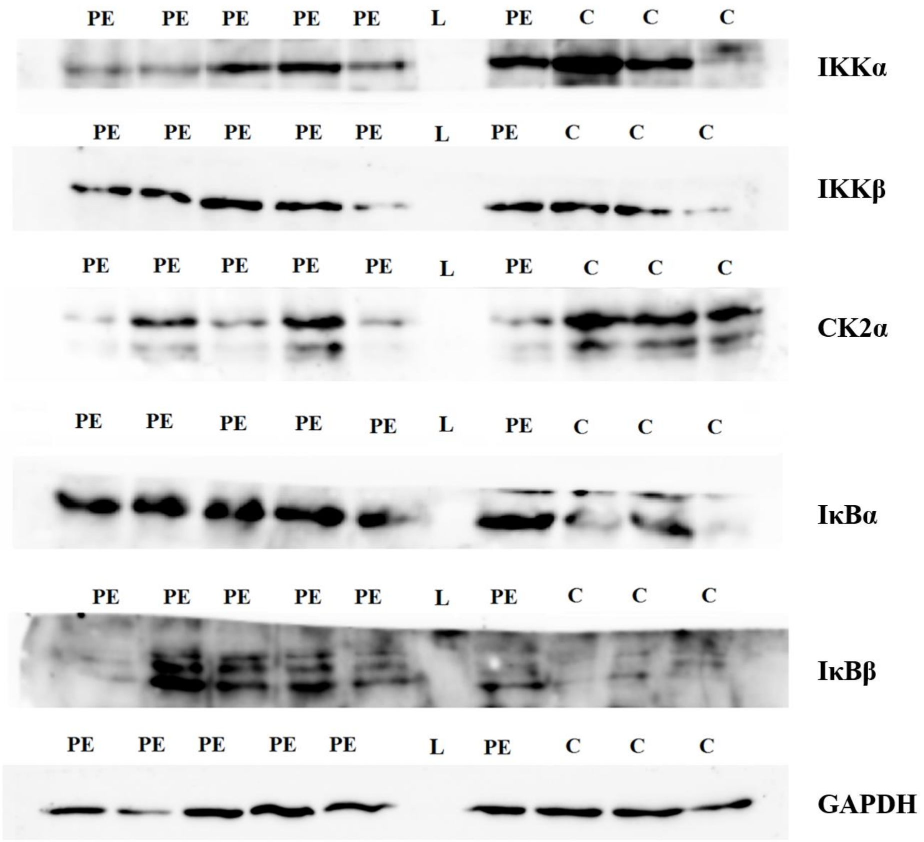

Supp. Fig. S1. Activators and inhibitors of NFκB in Western blot visualisation. Total protein fraction isolated from preeclamptic and control placentas were separated on the SDS-PAGE. After electrophoresis and semi-dry transfer, the PVDF membranes were divided according to the protein ladder. Each fragment was blocked and, in the next step, incubated with the one of the following antibodies: IKKα, IKKβ, CK2α, IκBα, IκBβ and GAPDH. The monoclonal anti-GAPDH antibody was used as internal control. PE – preeclampsia; C – control samples, L – line reserved for protein ladder.
